# Breaking Barriers in Student Mental Health Care With AI-Enhanced Group Cognitive Behavioral Therapy: Pilot Feasibility Study

**DOI:** 10.2196/84296

**Published:** 2026-06-12

**Authors:** Bogdan Tudor Tulbure, Bianca Spătaru, Andrei Rusu, Darian Faur, Daniel Precupaș, Loredana Marcela Trancă, Șerban Gabriel Popa, Adrian Spătaru

**Affiliations:** 1 Department of Psychology, West University of Timisoara, 4 Vasile Parvan Bvd, Timisoara, 300223, Romania, 40 745-753-061; 2 Institute for Advanced Environmental Research, West University of Timisoara, Timisoara, Romania; 3 Department of Computer Science, West University of Timisoara, Timisoara, Romania; 4 Department of Social Work, West University of Timisoara, Timisoara, Romania

**Keywords:** artificial intelligence, large language models, chatbots, university students, transdiagnostic, cognitive behavioral therapy

## Abstract

**Background:**

University students experience elevated psychological distress, with limited access to mental health services. While cognitive behavioral therapy (CBT) demonstrates efficacy for anxiety and depression, treatment gaps persist due to access barriers and insufficient between-session support. Large language model (LLM) chatbots could improve and scale CBT delivery. However, the scientific evaluation of chatbot-enhanced protocols is just emerging.

**Objective:**

This pilot study aimed to assess the feasibility, acceptability, and preliminary efficacy of an LLM-based ChatBot as an adjunct to group Unified Protocol (UP) therapy for between-session support in university students with subclinical anxiety and depression symptoms.

**Methods:**

A single-arm feasibility trial recruited university students aged 18 years and older with moderate subclinical symptoms (Social Phobia Inventory: 21‐40, Patient Health Questionnaire-9: 5‐14, or Generalized Anxiety Disorder-7: 5‐14), excluding those with current psychiatric disorders, suicidal ideation, or psychotropic medication use. The intervention comprised 4 weekly group UP counseling sessions complemented by an adjunctive Claude 3.7-Sonnet LLM ChatBot programmed with UP-based therapeutic prompts for between-session support rather than a stand-alone therapeutic agent. Primary feasibility outcomes included treatment adherence, chatbot engagement metrics, and system usability (System Usability Scale). Secondary outcomes assessed changes in generalized anxiety (Generalized Anxiety Disorder-7 Scale), social anxiety (Social Phobia Inventory), depression (Patient Health Questionnaire-9), and well-being (Short Warwick-Edinburgh Mental Wellbeing Scale) using paired *t* tests. Qualitative feedback was collected through focus group interviews and analyzed using thematic analysis.

**Results:**

Of 72 screened participants, 37 met eligibility criteria and 19 initiated treatment (mean age 22.06, SD 1.78 years; 70.6% female). Retention was high with 17 completers (10.5% dropout rate). Among completers, 94.1% (16/17) attended ≥3 group sessions. The engagement with the CBT ChatBot was substantial: participants were active on a median of 23 days during the 34-day study period and exchanged a median of 15 messages in total. System usability was rated as excellent (mean 84.94, SD 10.98 out of 100). Pre-to-post comparisons revealed significant improvements in generalized anxiety (mean change −3.00, SD 3.46; *t*_16_=3.01, *P*=.004; Cohen *d*=0.71) and mental well-being (mean change +2.29, SD 3.65; *t*_16_=−2.17, *P*=.02; Cohen *d*=0.69). Social anxiety and depression showed nonsignificant trends toward improvement. Qualitative feedback highlighted the CBT ChatBot’s accessibility and nonjudgmental support while noting limitations in personalization. No adverse events or inappropriate chatbot interactions occurred.

**Conclusions:**

Augmenting a group UP therapy with an LLM ChatBot demonstrated high feasibility, acceptability, and preliminary efficacy signals for university students with subclinical symptoms. The hybrid intervention package achieved strong retention and engagement while maintaining safety. These findings support progression to a randomized controlled trial to definitively evaluate this technology-enhanced approach for expanding access to evidence-based mental health interventions.

## Introduction

### Background

The global prevalence of anxiety and depression has created an urgent need for accessible, scalable mental health interventions. University students represent a particularly vulnerable and underserved group, facing elevated psychological distress and multiple structural barriers to mental health care [1,2]. Nearly 1 in 5 university students report moderate to severe anxiety or depression each year, and yet more than half never access professional care. In fact, among 18‐ to 25-year-olds who experienced a major depressive episode from 2011 to 2019, 53% did not receive any treatment in the past year [3].

Despite the robust empirical foundation of cognitive behavioral therapy (CBT) for the treatment of anxiety and depressive disorders [4,5], a substantial proportion of individuals, especially those in subclinical populations, do not fully benefit from traditional formats due to poor treatment adherence, limited access to mental health services, and insufficient personalization of therapeutic content [6,7]. Therefore, CBT does not yield therapeutic benefits for all individuals. Moreover, a comprehensive meta-analysis encompassing 409 randomized controlled trials (RCTs) reported that approximately 58% of patients fail to achieve a clinically significant response to CBT [8]. Several factors have been involved in this limited efficacy, with one of the most outstanding being the insufficient engagement with between-session therapeutic tasks, such as homework assignments designed to reinforce core treatment principles [9]. To ensure good efficacy, the intervention should facilitate engagement with between-session activities, such as completing therapeutic materials and exercises, consolidating the therapeutic gains, and supporting the generalization of skills made in the therapy to everyday contexts, thereby promoting both short-term progress and long-term improvements [10]. Therefore, fostering sustained, high-quality engagement with CBT exercises and materials between sessions represents a central aim to optimize treatment outcomes. This objective is particularly critical in group therapy contexts, where attrition rates tend to be higher and clinical recovery rates lower compared with individual therapy formats [11].

In recent years, artificial intelligence (AI)–driven “chatbot” therapy has emerged as a promising solution to bridge gaps in mental health care access and to strengthen between-session support, using integrating digital technologies. Early therapy chatbots were typically rule-based or scripted (eg, Woebot and Wysa) and demonstrated the capacity to deliver highly personalized user experiences [12], holding small but significant reductions in anxiety and depressive symptoms [13,14]. A 2024 meta-analysis [15] of 18 RCTs found that such AI-based chatbots produced modest short-term improvements in depression and anxiety, although effects often waned by 3-month follow-up. However, the advent of large language models (LLMs)—such as GPT-3, GPT-4, and similar generative AI systems—has enabled a new generation of chatbots capable of more natural, dynamic conversations. These LLM-based chatbots can flexibly generate responses and potentially assist clients in applying various psychotherapy techniques in a more human-like manner than rule-based predecessors.

Recently, research has shown that humans have difficulty differentiating responses written by a machine or a human, supporting the sentiment of Turing’s prediction [16]. Since chatbot outputs seem very similar to human outputs, this may serve as an early indication that LLMs have the potential to improve psychotherapeutic processes. Furthermore, AI-driven “chatbots” could enhance user engagement and promote greater adherence to therapeutic protocols [17], reinforcing patient involvement with therapeutic content outside of scheduled sessions. Moreover, chatbots offer the advantage of facilitating an open environment for those hesitant about seeking mental health assistance and guidance [18], allowing for more flexible conversations [19], enabling a higher degree of personalized support, and assisting the therapeutic process between sessions [20].

Although the effectiveness and safety of chatbots for mental health have been assessed in some studies [18], a definitive conclusion could not be drawn. In a systematic review and meta-analysis [21] examining the effect of using chatbots in mental health, the studies showed conflicting results for certain outcomes, such as positive and negative affect and anxiety. Additionally, the results revealed a high risk of bias in the evidence, characterized by significant methodological weaknesses and small sample sizes, which could explain the findings. Another limitation of LLM chatbots for mental health is that most models focus on active listening and problem exploration and do not capture the full and diverse range of therapeutic techniques [20]. Moreover, it is not clear whether chatbots are able to establish a genuine therapeutic alliance and communicate nuanced empathy [22]. The current literature also highlights the risk of overreliance and overdependence on trained and untrained chatbots for mental well-being support, as AI chatbots can appear reassuring and always available, leading users to turn to them instead of seeking professional help [23,24]. This false sense of sufficient support can cause people to postpone therapy, potentially allowing their disorder to worsen.

Although interest in AI-powered mental health services, particularly LLM-based chatbots, has surged since 2022, only a small number of these interventions have been evaluated through RCTs to date. The vast majority of AI mental health initiatives remain at the pilot or feasibility stage, characterized by small samples, single-arm or observational designs, and brief follow-ups (eg, the 21-Day Stress Detox trial, EMMA [EMotionAware mHealth Agent] emotion-aware chatbot, Wysa real-world evaluation, and Woebot postpartum pilot). However, these studies generally report good usability and user satisfaction, alongside modest improvements in symptoms of anxiety, depression, or stress. A summary of representative studies is presented in Table 1.

**Table 1.** Representative studies evaluating chatbot-based mental health interventions.

Study	Chatbot	Therapeutic approach	Study design	Sample	Duration	Key findings
Williams et al [25]	21-Day Stress Detox	Stress management	Open feasibility trial	Young adults from the general population experiencing stress (N=124)	21 days	High engagement and satisfaction; significant reductions in stress and anxiety
Ghandeharioun et al [26]	EMMA (EMotionAware mHealth Agent)	Emotion-aware just-in-time microinterventions	Feasibility study with control condition	Adults recruited from the general population using smartphones (N=39)	14 days	High usability and engagement; context-aware interventions increased acceptability
Sabour et al [27]	Emohaa	CBT^a^-based exercises + emotional venting	Pilot controlled study	Adults reporting psychological distress (N=134)		Significant reductions in anxiety and depression in chatbot groups
Melo et al [28]	ChatGPT conversational support	Conversational emotional support	Pilot feasibility study	Psychiatric inpatients receiving hospital treatment (N=12)	3‐6 sessions	Feasible and acceptable; improvements in quality of life reported
Fitzpatrick et al [29]	Woebot	CBT	Randomized controlled trial	College students with symptoms of depression and anxiety (N=70)	2 weeks	Significant reductions in depression compared with control
Klos et al [30]	Tess	CBT-based psychological support	Pilot randomized controlled trial	College students with anxiety symptoms (N=181)	8 weeks	Good acceptability and reductions in anxiety
Suharwardy et al [31]	Postpartum support chatbot	CBT-informed mood management	Randomized controlled trial	Postpartum women experiencing depressive symptoms (N=192)	6 weeks	Moderate improvements in depression compared with usual care
MacNeill et al [32]	Wysa	CBT-based coaching	Randomized controlled trial	Adults with chronic medical conditions (N=68)	4 weeks	Improvements in anxiety and depression
He et al [33]	XiaoE	CBT-based conversational therapy	Single-blind 3-arm randomized controlled trial	College students with depressive symptoms (N=148)	1 week + 1-month follow-up	Significant reductions in depression vs control chatbots

a CBT: cognitive behavioral therapy.

A recent bibliometric study [34] found that research on chatbots in mental health has been growing at approximately 46% per year, reflecting dozens of new prototypes across various platforms, each initially tested in small feasibility trials. This study underscores the idea that although RCTs for AI-powered mental health services are emerging, we are still in the early stages of development.

### Study Rationale and Objectives

To date, very few studies have systematically examined how AI chatbots could be integrated with existing evidence-based interventions, particularly in the context of group-based Unified Protocol (UP) [35,36]. Given the preliminary nature of the current evidence base and the absence of systematic evaluations integrating LLM-based chatbot support within established CBT protocols such as the UP, a full-scale RCT would have been premature. A feasibility study seemed, therefore, warranted to assess acceptability, usability, engagement, and preliminary outcome signals before advancing to a larger, definitive trial.

This study aims to address this gap by evaluating the feasibility of a hybrid intervention that combines a structured group counseling program (UP) with an LLM-based ChatBot that was available for participants between counseling sessions. This study specifically targets university students with subclinical symptoms of anxiety and/or depression, a population known to experience elevated psychological distress and limited access to traditional mental health services. By embedding a CBT ChatBot into a well-validated protocol, this study leverages structured emotion regulation strategies while using AI to reinforce and personalize homework assignments between meetings. The decision to adopt this approach was guided by recent literature highlighting the potential of hybrid health care chatbots to increase engagement and optimize treatment outcomes.

In this study, we collected both quantitative metrics (eg, session attendance, chatbot-user interaction rates, and outcome data) and qualitative feedback (through a focus group interview at the end of the program) to develop a richer feasibility profile. This design also aligns with the Medical Research Council framework for complex intervention development, which emphasizes feasibility testing before proceeding to full-scale RCTs [37]. Moreover, the current trial serves as a critical exploratory function, such as allowing for the calibration of chatbot functionality within a defined therapeutic framework, providing qualitative insights into user experience, barriers, and generating preliminary data on symptom change and engagement patterns that are essential for designing future RCTs. Importantly, this study also contributes to emerging research on how AI tools can ethically and effectively augment—but not replace—human-delivered psychological counseling, especially in low-resource and high-demand settings such as universities. In sum, this study fulfills a dual scientific requirement: (1) to explore the integration of cutting-edge LLM technology within evidence-based psychological care and (2) to empirically ground the scalability of such interventions through rigorous feasibility evaluation prior to broader implementation.

Specifically, this study’s objectives include (1) evaluating user acceptability, usability, and satisfaction with the CBT ChatBot; (2) investigating patterns of interaction between sessions with the CBT ChatBot; (3) assessing retention and engagement rates with the hybrid intervention package; (4) exploring preliminary changes in anxiety, depression, and well-being; and (5) identifying barriers and facilitators to implementing this hybrid model in a university counseling context.

## Methods

### Ethical Considerations

This study was reviewed and approved by the Scientific Council of University Research and Creation of the West University of Timișoara (approval no. 22170, dated March 31, 2025). All procedures were conducted in accordance with the ethical standards of the relevant institutional and national research committees and with the 1964 Helsinki Declaration and its later amendments. Prior to participation, all participants provided written informed consent. Participant data were anonymized and stored securely in accordance with applicable data protection regulations.

### Procedure

This study is a single-arm, uncontrolled intervention trial. For accessibility reasons, a convenience sampling method was used. A total of 37 university students were selected after the screening (April 2025). Participants were recruited via online announcements on the authors’ (personal) and the university’s (public) social media outlets. Interested participants read and electronically signed the informed consent. Subsequently, they were invited to complete a series of self-report measures and demographic characteristics as part of the screening procedure.

The inclusion criteria were the following: (1) at least 18 years of age, (2) at least 1 self-report measure showing moderate symptoms of anxiety and/or depression (ie, between 21 and 40 for Social Phobia Inventory, 5 and 14 for Patient Health Questionnaire-9 [PHQ-9], and 5 and 14 for Generalized Anxiety Disorder-7 Scale [GAD-7]), (3) no current psychological disorder (according to demographics and medication status), (4) no suicidal ideation (ie, not to exceed a score of 1 on the PHQ-9 suicide item), (5) no psychotropic medication, and (6) currently not taking part in any other psychosocial interventions.

Eligible participants were invited to the group counseling sessions, while ineligible participants received a message with the reason for exclusion and, if appropriate, suggestions for potentially relevant resources. The intervention program consisted of 4 face-to-face group counseling sessions organized once per week in May 2025. Participants could choose 1 of the 3 time slots for the small group counseling sessions based on their schedule or preferences. At the end of the first in-person meeting, participants were provided access to the CBT ChatBot. They were informed that the ChatBot was designed for independent use between weekly sessions, as a self-directed support tool to reinforce session content.

A PhD-level clinician and 3 graduate students in clinical psychology served as group facilitators, providing guidance and support to participants throughout the intervention. The facilitators completed a structured 4-hour training course covering the UP’s core principles, session content, and delivery procedures under the supervision of an experienced psychotherapist. Moreover, the PhD clinician had prior clinical experience implementing the full UP protocol with clinical cases during the graduate training, further strengthening the team’s applied expertise with the model.

At the end of the program (ie, after 4 weeks), all participants were invited to complete the postintervention assessment measures. They were also informed that they could volunteer for the focus group that was organized during week 5 in order to document the facilitating factors and barriers encountered during implementation. All participants (n=14) provided informed consent after being briefed on the focus group’s purpose and assured of confidentiality. The session was audio-recorded and lasted for 1 hour. The moderator, a researcher (LMT) with CBT background, guided the interview in Romanian, fostering an environment where participants felt comfortable sharing their individual perspectives, without feeling pressure to agree with the other participants. Relevant qualitative data extracted and analyzed from the focus group, including illustrative examples, were translated into English after the finalization of the coding scheme and data interpretation. Participants were informed that they would be financially compensated, although the exact amount was not communicated in advance. They received modest compensation of €10 (€10 = US $11.09) for each face-to-face counseling session attended, including the focus group, as well as for completion of the postintervention assessment. Compensation was not contingent on symptom improvement or favorable responses. To minimize the potential influence of financial incentives on willingness to participate, payments were disbursed only after completion of the study.

### Measures

#### Feasibility and Usability Measures

Treatment adherence was estimated through the number of completed therapy sessions and the dropout rate (number of participants who did not complete the postintervention assessment).

CBT ChatBot’s usage frequency was operationalized through the (1) number of days of activity during the program (out of 34 possible maximum days and relative to 28 during the unfolding of the therapy sessions), (2) total number of conversations with the CBT ChatBot, (3) total number of conversations shared with the therapist, (4) total number of reported conversations as inappropriate, and (5) total number of messages sent to the CBT ChatBot (across all conversations).

System Usability Scale (SUS) [38,39] was used to assess the overall experience participants had with the CBT ChatBot. SUS includes 10 items designed to capture the effectiveness, efficiency, and satisfaction of the user with the system, and each item is scored on a 5-point Likert scale (ranging from 1 “strongly disagree” to 5 “strongly agree). The reported internal consistency for this study was optimal (α=.91).

The focus group interview: During the focus group (semistructured interview), we used open-ended questions organized around 4 core themes: general experience with the program, facilitating factors for successful implementation, barriers and difficulties encountered during implementation, and suggestions for program improvement.

#### Outcome Measures

Social Phobia Inventory [40] includes 17 items designed to measure social anxiety symptoms. The instrument returned excellent internal consistency indices, ranging from α=.87 to .94 across different groups [41]. The scale performed similarly in our sample, with α=.87.

GAD-7 [42] was used to measure worry symptoms. It uses a Likert scale from 0 (“not at all”) to 3 (“nearly every day”). The internal consistency of the scale was optimal (α=.92). In our sample, the baseline internal consistency for GAD-7 was α=.70.

PHQ-9 measures depression symptoms in a concise format. It is a widely used instrument using the same 4-point Likert scale as previously mentioned, with an adequate internal consistency index (α=.89) [43]. PHQ-9 returned a similar internal consistency index in our sample (α=.84).

Short Warwick-Edinburgh Mental Wellbeing Scale is a 7-item instrument derived from a larger questionnaire that measures mental well-being using a 5-point Likert scale that ranges from 1 (“none of the time”) to 5 (“all of the time”). It presented adequate internal consistency (α=.84) [41,44]. Regarding our sample, the scale presented acceptable internal consistency of α=.77.

### The Intervention Program and Chatbot Application

#### Overview

*The psychosocial intervention* was based on the Unified Protocol for Transdiagnostic Treatment of Emotional Disorders [35]. The core therapeutic principles were adapted for a counseling-oriented intervention designed to address subclinical symptoms of anxiety and/or depression. Drawing on prior clinical experience with the UP [45,46], we condensed the protocol into a 4-session version that preserved its core therapeutic principles while eliminating explicit diagnostic terminology or disorder-specific content. The counseling program presented the UP strategies as generalizable skills for understanding and managing emotions in everyday life, thereby increasing accessibility and reducing potential stigma.

#### Session 1: Understanding Emotions Through the Tripartite Model

Participants were introduced to the tripartite model of emotions, emphasizing the interplay of physiological arousal, cognitive appraisal, and behavioral responses in emotional experiences. This module fostered awareness of their own emotional responses and patterns, as well as the role of both adaptive and maladaptive strategies. Motivational enhancement strategies were incorporated to clarify individual goals for emotional self-regulation and treatment engagement.

#### Session 2: Cognitive Reappraisal and Flexible Thinking

Building on emotional awareness, participants were taught to identify and modify *cognitive distortions* that influence emotional intensity and behavioral responses. This component emphasized cultivating cognitive flexibility and adopting alternative, more adaptive interpretations of emotional triggers, consistent with the UP’s focus on adaptive appraisal.

#### Session 3: Emotion-Driven Behaviors and Behavioral Flexibility

This session targeted *emotion-driven behaviors*—actions that are primarily motivated by an attempt to avoid, escape, or control unwanted emotional states. Participants learned to identify these patterns and to engage instead in *opposite actions* and approach-oriented behaviors aligned with long-term values, thereby reducing avoidance and promoting adaptive functioning.

#### Session 4: Skills Consolidation and Relapse Prevention

The final module reviewed the key UP strategies learned throughout the program, reinforced their integration into daily routines, and developed individualized *relapse prevention* and maintenance plans. Participants identified early warning signs of emotional dysregulation and selected strategies to sustain gains over time.

By adapting the UP in this manner, the intervention sought to preserve its transdiagnostic, emotion-focused framework while delivering it in a brief and nonpathologizing counseling format, emphasizing emotional awareness, cognitive flexibility, and behavioral engagement as core change mechanisms.

*The CBT ChatBot web application* used Claude 3.7-Sonnet LLM, with system prompts explicitly embedding the core therapeutic principles of the UP. These UP-based prompts were designed to directly guide the chatbot’s responses, ensuring that all interactions were consistent with the model’s emotion-focused framework. Prior to deployment, the chatbot’s output was pretested by the 3 graduate students (SGP, AMC, and MS) in clinical psychology, who assumed the role of clients and engaged in simulated conversations. They provided structured feedback on the appropriateness, therapeutic consistency, and clarity of responses, and all suggested improvements were incorporated into the final chatbot design. The system prompt was further customized for each participant by including a concise summary of prior interactions to preserve conversational continuity. To optimize processing efficiency and reduce input token costs, the same LLM was used with a summarization prompt to condense conversation histories into brief summaries.

The CBT ChatBot’s environment was developed using state-of-the-art software standards and libraries to ensure data security and privacy. All participants accessed the intervention via a secure HTTPS connection to a university-hosted server. The platform architecture comprised a front end, back end, database, and external service integrations. The front end, delivered via the user’s web browser, allowed participants to interact with the chatbot and review archived conversations. The back end managed authentication, role assignments, message processing, and secure communication with the database and integrated services.

Participants accessed a dashboard displaying the CBT ChatBot’s interface and archived conversations, with options to share specific exchanges with their therapist or to flag conversations perceived as inappropriate or potentially harmful. This flagging functionality was implemented both to acknowledge the inherent limitations in controlling AI-generated content and to reinforce participant awareness that they were engaging with a chatbot rather than a human therapist. In line with a “human-in-the-loop” safety principle, therapists could review both shared and flagged or inappropriate conversations, with flagged cases receiving immediate attention. Administrators had access to usage statistics and permissions to create and assign user roles.

All conversation histories and summaries were encrypted using symmetric key cryptography, with a unique key assigned to each participant. Shared conversations were additionally encrypted using the therapist’s key. For security and operational efficiency, encryption keys were generated and stored on the server. In accordance with platform safety protocols, any inappropriate or flagged conversation was set to be automatically shared with the therapist for urgent review. However, during the trial no conversation was flagged as inappropriate.

### Data Analytic Plan and Study Power

Feasibility, usability, and satisfaction with the program were analyzed descriptively by reporting frequencies for categorical indicators and measures of central tendency and dispersion for continuous ones. Moreover, pre- to postintervention comparisons were conducted using 1-tailed paired samples *t* tests, since we expected that our intervention would produce improvements in the aimed outcomes (hence, we tested, on a confirmatory basis, whether our anticipated direction emerged), while between-group comparisons were analyzed using 2-tailed independent samples *t* tests, since these were purely exploratory without any a priori expectations for differences between adherent and nonadherent participants.

As this was a pilot feasibility study, sample size determination was based on feasibility objectives rather than statistical power calculations for efficacy outcomes. Following established guidance for pilot studies aiming to detect small effects [47], we targeted recruitment of 30‐40 eligible participants to provide sufficient data for assessing key feasibility parameters, including recruitment rates, retention, intervention adherence, and chatbot engagement patterns. This sample size was deemed adequate to evaluate the feasibility and acceptability of the intervention approach and generate preliminary effect size estimates for future sample size calculations in a definitive RCT. The final recruited sample (n=37 eligible; n=17 completers) meets these feasibility objectives.

The focus group interview was transcribed using Word online, and the transcription was manually reviewed by one of the research team members (SGP) to ensure accuracy. The data collected were anonymized and thematically analyzed using the guidelines proposed by Braun and Clarke [48,49]. To enhance trustworthiness, the data were inductively coded by 2 coders (LMT and SGP), members of the project team with backgrounds in social work and psychology (both with teaching experience). Each coder independently developed a thematic map of the analysis, identifying themes and categories. The analysis began with a phase of familiarization, during which each coder read the transcripts multiple times to gain an understanding of the participants’ accounts. Following this, the first iterative process was realized, where initial codes were generated inductively. Coding was conducted manually, without the use of qualitative data analysis software. These codes were refined during additional iterative rounds to better capture the nuances of the data. Each code was linked with corresponding transcript excerpts to ensure a transparent link between the analysis and the participants’ original voices. The 2 coders moved to theme development, and the codes were grouped into broader categories of meaning.

A peer debriefing session was then conducted; the coders engaged in a dialogic process to negotiate and harmonize these categories, resulting in a consolidated thematic map. At this stage, we noticed that the social worker’s background oriented her attention toward contextual, relational, and support system dimensions of students’ experiences, particularly in relation to accessibility and barriers perceived with the hybrid counseling model, while the psychologist’s background foregrounded psychological processes related to the perceived therapeutic value of the AI-supported components between sessions. Reflexive discussions were therefore integrated into the analytic process.

To ensure confirmability, a final stage of checking back was conducted, where both, 1 coder and 1 of the team members who participated in collecting data, reread the entire focus group transcript and the consolidated thematic map. This step served to verify that the identified themes were comprehensively supported by the raw data and were not merely a reflection of the researchers’ preconceptions. Subsequently, 1 coder reread the entire focus group transcript to confirm that the identified themes and categories were comprehensively supported by the raw data. A narrative approach was used to present the findings, emphasizing participants’ individual experiences as they relate to the research questions and identified themes.

## Results

### Participants

**Figure 1.**
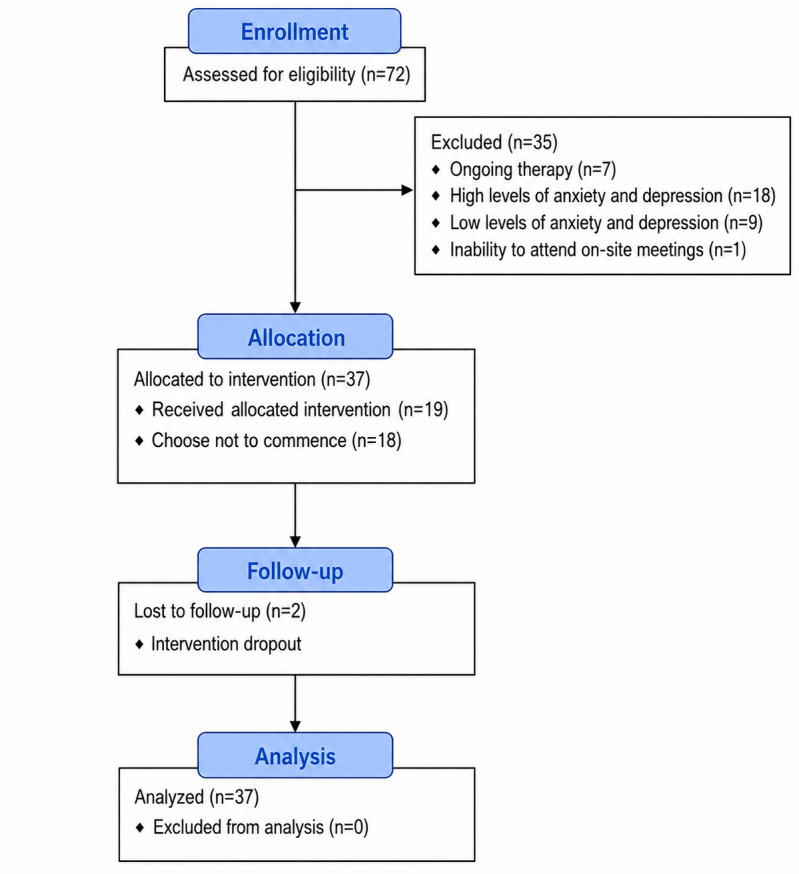
Modified CONSORT (Consolidated Standards of Reporting Trials) flow diagram for participants’ recruitment and progress throughout the program.

After the recruitment call was disseminated, 72 potential participants registered and were screened for eligibility. Of these, 37 met all eligibility criteria and were invited to take part in the counseling sessions. Ultimately, 19 participants responded and attended the first session, and 17 participated in the majority of the sessions and completed the postintervention measures (ie, completers; Figure 1). The completers had an average age of 22.05 (SD 1.80) years, with 70.6% (12/17) identifying as female. All were either first-year (9/17, 52.9%) or second-year (8/17, 47.1%) students. Overall, 41.2% (7/17) of the active participants had previously undergone psychotherapy, and 3 of these (3/17, 17.6%) reported a former psychiatric diagnosis (ie, anxiety, depression, or posttraumatic stress disorder). None were currently undergoing mental health treatment. The baseline demographic and clinical characteristics of the participants’ groups involved in this study can be consulted in Table 2.

**Table 2.** Baseline demographic and clinical characteristics in different groups.

Variable	Allocated to intervention (n=37)	Received the intervention (n=19)	Focus group (n=14)
Age (years), mean (SD)	23.24 (5.04)	22.05 (1.80)	22.42 (1.74)
Sex, n (%)			
Male	11 (29.7)	5 (26.3)	3 (21.4)
Female	26 (70.3)	14 (73.7)	11 (78.6)
Marital status, n (%)			
Never married	28 (75.7)	13 (68.4)	10 (71.4)
In a relationship	9 (24.3)	6 (31.6)	4 (28.6)
Study program, n (%)			
Undergraduate studies	26 (70.3)	14 (73.7)	8 (57.1)
Graduate studies	11 (29.7)	5 (26.3)	6 (42.9)
Previous psychotherapy (last 4 years), n (%)			
Yes	13 (35.1)	7 (36.8)	8 (57.1)
No	24 (64.9)	12 (63.2)	6 (42.9)
GAD-7^a^, mean (SD)	10.35 (3.47)	9.94 (3.25)	9.50 (3.39)
PHQ-9^b^, mean (SD)	12.56 (6.06)	11.63 (5.85)	12.07 (6.43)
SPIN^c^, mean (SD)	24.37 (10.67)	25.42 (12.09)	24.21 (12.73)
SWEMWBS^d^, mean (SD)	23.59 (3.91)	24.21 (4.15)	23.42 (3.69)

a GAD-7: Generalized Anxiety Disorder-7 Scale.

b PHQ-9: Patient Health Questionnaire-9.

c SPIN: Social Phobia Inventory.

d SWEMBWS: Short Warwick-Edinburgh Mental Wellbeing Scale.

Of the 19 participants who started the program, only 2 dropped out completely, resulting in an overall dropout rate of 10.5%. The majority of adherent participants (10/19, 58.8%) attended all 4 group sessions, while 35.3% (6/19) of the participants attended at least 3 sessions. Only 1 participant (5.9%) attended a single session. Thus, overall adherence to the group sessions was high.

Regarding the usage of the CBT ChatBot, engagement was frequent throughout the study. The maximum duration between account creation and the end of the study was 34 days, during which the group sessions took place over 28 days. During this time frame, participants were active on a median of 23 days. On average, participants initiated a median of 3 conversations with the CBT ChatBot, of which approximately 1 was shared with the therapist (in other cases, the CBT ChatBot’s’s input was deemed sufficient). None of the interactions were considered inappropriate or unsatisfactory. Across all conversations, participants exchanged a median of 15 messages. Overall, these data suggest a high level of engagement with the CBT ChatBot (see Table 3 for details). Moreover, the CBT ChatBot’s usability, as measured by the SUS [38], was rated as excellent (mean 84.94, SD 10.98; median 88 out of 100).

### Statistical Analysis

Data regarding the feasibility, usability, and the satisfaction with the intervention program are presented in Table 3. No participants reported inappropriate conversations with the CBT ChatBot during the study. As Table 4 shows, generalized anxiety symptoms and subjective well-being regarding mental health significantly improved from pre- to postintervention. These improvements were moderate to large in magnitude. However, neither social anxiety nor depressive symptoms recorded significant improvements—despite a trend in the expected direction.

**Table 3.** Frequency and scope of usage of the CBT^a^ ChatBot during the program implementation (N=17).

Variable	Minimum	Maximum	Mean (SD)	Median (IQR)
Days of activity during the program (maximum possible 34)	1	32	19.58 (10.21)	23 (18)
No. of conversations with the CBT ChatBot	0	26	4.35 (5.82)	3 (3)
No. of conversations shared with the therapist	0	26	1.82 (6.30)	0 (0)
No. of reported conversations as inappropriate (flagged)	0	0	0 (0)	0 (0)
Total messages sent to the CBT ChatBot (across all conversations)	0	197	40.35 (59.34)	15 (27)

a CBT: cognitive behavioral therapy.

**Table 4.** Pre- to postintervention comparisons for each mental health outcome (N=17).

Variable	Pre, mean (SD)	Post, mean (SD)	*t* test (*df*)	*P* value^a^	Cohen *d*^b^ (95% CI)
GAD-7^c^	10.18 (3.36)	7.18 (4.76)	3.01 (16)	.004	0.71 (0.19 to 1.22)
SPIN^d^	26.35 (12.38)	23.06 (13.60)	1.07 (16)	.15	0.25 (−0.22 to 0.73)
PHQ-9^e^	12.59 (5.41)	10.65 (6.60)	1.24 (16)	.12	0.32 (−0.20 to 0.84)
WEMWBS^f^	23.59 (3.84)	25.88 (2.74)	–2.17 (16)	.02	0.69 (0.01 to 1.37)

a *P* values are 1-tailed.

b Cohen *d* effect size estimate.

c GAD-7: Generalized Anxiety Disorder-7 Scale.

d SPIN: Social Phobia Inventory.

e PHQ-9: Patient Health Questionnaire-9.

f WEMWBS: Warwick-Edinburgh Mental Wellbeing Scale.

To examine potential attrition bias, we compared participants who did not initiate the study or withdrew before completion (n=20) with those who fully completed the study protocol (n=17), finding no significant differences between groups on any of the 4 outcome measures, age, or gender (Table 5). Therefore, there appears to be no discernible pattern distinguishing participants who chose not to adhere to the intervention, despite having initially intended to and being deemed eligible.

**Table 5.** Between-group comparisons of eligible participants who did not initiate the intervention and those who did.

Variable	Nonadherent (n=20), mean (SD	Adherent (n=17), mean (SD)	*t* test (*df*)	*P* value^a^	*d*^b^ (95% CI)
Age (years)	24.25 (6.58)	22.06 (1.78)	1.33 (35)	.19	0.43 (−0.21 to 1.09)
GAD-7^c^	10.50 (3.65)	10.18 (3.36)	0.28 (35)	.78	0.09 (−0.55 to 0.73)
SPIN^d^	22.70 (8.97)	26.35 (12.38)	−1.04 (35)	.31	−0.34 (−0.99 to 0.31)
PHQ-9^e^	12.55 (6.72)	12.58 (5.41)	−0.02 (35)	.99	−0.00 (−0.65 to 0.64)
WEMWBS^f^	23.60 (4.08)	23.58 (3.84)	0.01 (35)	.99	0.00 (−0.64 to 0.65)

a *P* values are 2-tailed.

b Cohen *d* effect size estimate.

c GAD-7: Generalized Anxiety Disorder-7 Scale.

d SPIN: Social Phobia Inventory.

e PHQ-9: Patient Health Questionnaire-9.

f WEMWBS: Warwick-Edinburgh Mental Wellbeing Scale.

### Qualitative Analyses of the Intervention Program

During the postintervention focus group interview, participants frequently highlighted the readily available and permanent support offered by the CBT ChatBot. This underscores the Bot’s role in providing immediate support outside the conventional therapy program: “...I had some more... anxious or pessimistic thoughts at midnight... And then I always had the chance to send a message and receive a reply”; “I only used it in the evening, at night, when you can’t go to a therapist.” Additionally, the CBT ChatBot provided a sense of safety, allowing users to express themselves without fear of being judged: “It was much easier for me to be vulnerable through messages with the CBT ChatBot than in the group sessions, because I didn’t know the people well and I didn’t feel comfortable enough to express myself freely.” “I found it easier to open up to the CBT ChatBot than during group interventions, because I didn’t feel judged...I knew it was just a program and couldn’t make judgments.” This perception of being free from judgment allowed participants to be more vulnerable than they might have been in on-site interactions. Beyond mere accessibility, the CBT ChatBot demonstrated usefulness by providing practical support and fostering a sense of comfort among users. A participant shared: “I interacted with it when I had sort of a panic attack and wanted to calm down. (...) And it really helped me calm down quite well.” This illustrates the Bot’s capacity to offer immediate, effective solutions during crises or vulnerable moments. The ability of the CBT ChatBot to provide pertinent advice and its structured approach to feedback was perceived as beneficial, with one respondent remarking: “I felt like I was talking to a friend. And I even noticed a useful dissection and analysis of the situation, greater than a friend would have offered.”

However, some participants also expressed dissatisfaction with the CBT ChatBot’s impersonal nature and repetitive response patterns. A recurring sentiment among participants was the Bot’s lack of empathy, like one user stating: “...I didn't feel a certain degree of empathy and real connection with it.” This highlights a disconnection experienced by some users who sought a more human-like, nuanced interaction that the AI could not provide. Furthermore, the content provided by the CBT ChatBot was frequently perceived as generic or repetitive; participants encountered recurring advice that did not evolve based on prior input (such as repetitive suggestions to “Breathe!”), which led to frustration and disengagement: “I didn’t really like the chatbot. (...) I stopped using it because it felt very impersonal.” Another barrier reported was related to the perceived superficiality and lack of customization in CBT ChatBot’s responses. Some participants described the feedback as “banal” and insufficiently tailored to their emotional complexity. Beyond content relevance and perceived impersonality, participants reported practical issues related to the Bot’s interface and functionality. Technical errors and complex login procedures were a source of frustration. “It was very annoying and frustrating that we had to log in with that password we received every time.” This administrative hurdle discouraged consistent engagement. Additionally, participants noted the Bot’s limitations in maintaining context across conversations and its tendency to ask repetitive questions: “...it gave general details and asked me again about the same things I had already told it, without it knowing it was a new conversation.” Participants also expressed concerns that an excessive reliance on the CBT ChatBot could result in a reduced capacity for independent coping or an overreliance on technology for emotional support. “Maybe some people would get very attached and become dependent on it...” Furthermore, participants expressed broader anxieties about the future of human interaction in an AI-dominated world, as one respondent explained: “I’m afraid that in the future people would really become dependent on AIs and we wouldn’t talk to the people around us anymore; that is, we would all focus strictly on technology.”

Another relevant aspect is related to the implicit comparison between the CBT ChatBot and the Small Group social interactions that were part of this hybrid intervention program. Participants expressed a proclivity for the group sessions, emphasizing the value of direct human interaction, which provided an emotionally supportive environment, as one respondent articulated: “I preferred face-to-face group meetings because it’s different when I sit in front of a computer and simply tell it my thoughts, I know the whole process behind it, that it’s built to give certain answers, but when I talk to a person is different.” Beyond the facilitator’s role, participants emphasized the therapeutic effect of engaging with other students who face similar challenges, with these interactions proving to be a source of emotional well-being: “I really liked the chatbot... but I think the group meetings helped me much more, because I was able to talk and be honest that I have a problem and it’s affecting me, and I can’t cope. And as I was talking, I became calmer (in the group).” This comment underscores the importance of face-to-face communication and the unique, irreplaceable empathy and understanding that can be provided by humans. Moreover, the psychoeducational value of the group sessions was also emphasized. Participants reported learning practical emotional regulation strategies and appreciated the structured, informative nature of the group meetings. A respondent noted: “...it was more like a kind of course... about how we regulate our emotions and how we apply these tools in our personal lives.” Despite these advantages, the group sessions were not without initial challenges. Participants reported discomfort with self-disclosure in a group context and occasional misalignment between their expectations and the session format. These findings highlight inherent complexities in group dynamics, particularly the need for some individuals to establish greater trust and familiarity before engaging in personal sharing.

Finally, participants identified several improvement strategies for the CBT ChatBot across multiple domains: interaction personalization, functional enhancement, interface design optimization, user-guided training mechanisms, and privacy and cybersecurity protocols. One participant proposed: “...a quiz should be given for each user.” The user should choose, “Look, I want you to usually answer like this, I want you to do these things, I want you to answer using that kind of language...” This highlights users’ preference for tailoring AI’s communication style and content to individual needs, potentially based on initial assessments and expressed preferences. In terms of the Bot’s functionality, common suggestions included incorporating voice interaction features (voice recognition and auditory responses to speech) for a more natural conversational flow: “I would have liked to receive some auditory answers...” Furthermore, there were frequent mentions of the need for an improved user interface, including dark and light mode options, as well as more intuitive button placement, to enhance overall usability. Other interface-related suggestions included simplifying the login process to improve accessibility. Although some participants suggested adding features such as (1) avatar assistance, (2) user progress tracking, or (3) follow-up messages asking whether prior discussion topics had been fruitful or not, opinions were divided. Some viewed these features as supportive, while others found them intrusive. Participants also raised some considerations regarding data security and ethical stewardship. Recommendations centered around implementing more transparent privacy policies and enhancing user autonomy over personal data. Finally, participants expressed concerns about potential psychological dependency on AI-mediated interventions, suggesting that access to the CBT ChatBot is beneficial, but it should be used adequately and in conjunction with more traditional strategies for emotion regulation. Table 6 provides a summary of the thematic map used for the focus group.

**Table 6.** Thematic map of the analysis of data from the focus group.

	Facilitators	Barriers	Proposals for improvement
CBT^a^ ChatBot experience	Accessibility and permanent availability Safe and nonjudgmental perceived free space Usefulness and concrete results Sense of comfort	Lack of empathy and real connection Content and relevance issues Interface and functionality issues Risk of dependency	Personalizing interaction Improving CBT ChatBot functionality Improving interface design Training the program by the users Ethical considerations Privacy and cybersecurity options
Group sessions experience	Human interaction and social support Educational and informational value	Reluctance to speak openly	

a CBT: cognitive behavioral therapy.

## Discussion

### Main Findings

Our pilot results indicate that integrating an LLM-based “CBT ChatBot” into UP therapy was broadly feasible and well received. The CBT ChatBot was designed to reinforce the in-person session content and to provide psychoeducational support between weekly group counseling sessions. Students engaged actively with the ChatBot throughout the 4-week program, using it to discuss emotions and practice UP skills between sessions. Attrition was low, and qualitative feedback reflected high acceptability—participants frequently described the AI companion as helpful, supportive, and easy to talk to. This mirrors emerging evidence that users often form a positive working alliance with therapeutic chatbots. In a recent RCT of a fine-tuned LLM therapist (“Therabot”), users’ ratings of empathy, understanding, and trust in the AI were “comparable to that of human therapists,” with many experiencing the chatbot as a genuinely caring presence [50]. Similarly, Kang and Hong [51] tested a GPT-4 chatbot and found very high user satisfaction, with average scores around 9/10 for feeling “supported” and approximately 8.7/10 for the bot’s empathy. Notably, participants in their study felt that the AI was encouraging, understanding, and a good listener, and no users reported feeling misunderstood or upset by the chatbot. Our findings align with these reports—students appeared comfortable confiding in the chatbot and felt “heard” by it, suggesting that even without human reciprocity, an LLM can foster a meaningful sense of rapport. Together, these results seem to support the idea that AI systems trained to incorporate psychotherapy principles could be helpful for users and challenge the assumption that a therapeutic alliance requires a human provider. Users seem able to benefit from AI-mediated digital support tools, at least for milder problems, likely because modern chatbots excel at conveying empathy and responsiveness. That said, some nuances of alliance merit caution. Moore et al [52] argue that an LLM-based “therapist” with generic training (ie, that was not specifically trained to follow therapeutic principles) may only mimic empathy and could foster a superficial alliance by telling clients what they want to hear, without the authenticity or judicious pushback of a human clinician. However, in this study, the CBT ChatBot played only an adjunct role, complementing the group counseling sessions to mitigate these concerns. Participants knew that their human counselor was overseeing all activities, while the CBT ChatBot’s role was to reinforce skills and provide support between sessions. This collaborative setup likely helped maintain trust, as the AI system was an extension of the therapy team. Overall, the high acceptability (with several students indicating that they would hypothetically “recommend the CBT ChatBot to a friend”) supports the feasibility of blending LLM chatbots into clinical care.

As an exploratory pilot, our trial was not powered to definitively test clinical efficacy. Nevertheless, the preliminary outcomes are encouraging. Students who were granted access to the CBT ChatBot alongside the UP group counseling sessions showed greater reductions in anxiety and significant improvements in their well-being levels. These results—while modest in absolute terms—suggest that the CBT ChatBot may add value by reinforcing the UP techniques and by encouraging emotional processing between sessions. Importantly, our findings align with early evidence that LLM-driven chatbots can produce meaningful short-term symptom relief. Yokotani et al [53] demonstrated in an RCT that a stand-alone UP-based chatbot led to significantly greater anxiety reduction in university students than in a waitlist control, with benefits sustained at 4-week follow-up. Notably, the chatbot group in that study also exhibited more frequent expressions of sadness and other negative emotions during the intervention, supporting the proposed mechanism that encouraging emotional expression can facilitate symptom reduction. This resonates with our qualitative observations—students commonly used the CBT ChatBot to vent frustration or sadness in a safe space, consistent with UP principles of emotional exposure and acceptance. Other recent trials underscore that LLM chatbots can yield clinically relevant improvements. For instance, Heinz et al [50] reported large effect sizes (Cohen *d* approximately 0.8‐0.9) for symptom reduction in depression and anxiety disorders after just 4 weeks of AI-guided therapy alone, an impact approaching that of in-person psychotherapy. Meanwhile, a smaller RCT by Chen et al [54] found that an AI chatbot’s immediate effectiveness in reducing mild anxiety or depression was statistically noninferior to a live mental health counselor (ie, nurse hotline group), with both yielding significant pre-post symptom reductions. In aggregate, these studies suggest that for common mental health concerns, LLM chatbots can achieve at least short-term efficacy on par with established interventions—especially as adjuncts or early support tools. On the other hand, broader analyses temper this optimism. A meta-analysis by Zhong et al [15] covering 18 trials of mental health chatbots found only modest overall effects (ie, Hedges *g*=0.19-0.26 for anxiety and depression). These benefits often diminished by 3-month follow-ups, indicating that chatbot gains may be less durable without continued use. Our pilot’s positive outcomes should therefore be interpreted in light of these modest averages and the subclinical nature of our sample. Indeed, our qualitative data indicated improvements in coping and emotional insight more so than dramatic symptom remission. This is consistent with the idea that LLM chatbots may be best suited for mild to moderate distress and skill practice, rather than as a sole treatment for severe disorders. Overall, our efficacy signals align with the emerging literature: LLM chatbots show real but preliminary potential as adjunctive tools for skills reinforcement and emotional processing between formal sessions. They appear capable of delivering evidence-based techniques (such as cognitive reappraisal or mindfulness prompts) in ways that measurably alleviate distress in the moment. The task ahead is to verify these effects in fully powered trials and to determine how sustained and generalizable the improvements can be.

### Safety and Ethical Considerations

**Figure 2.**
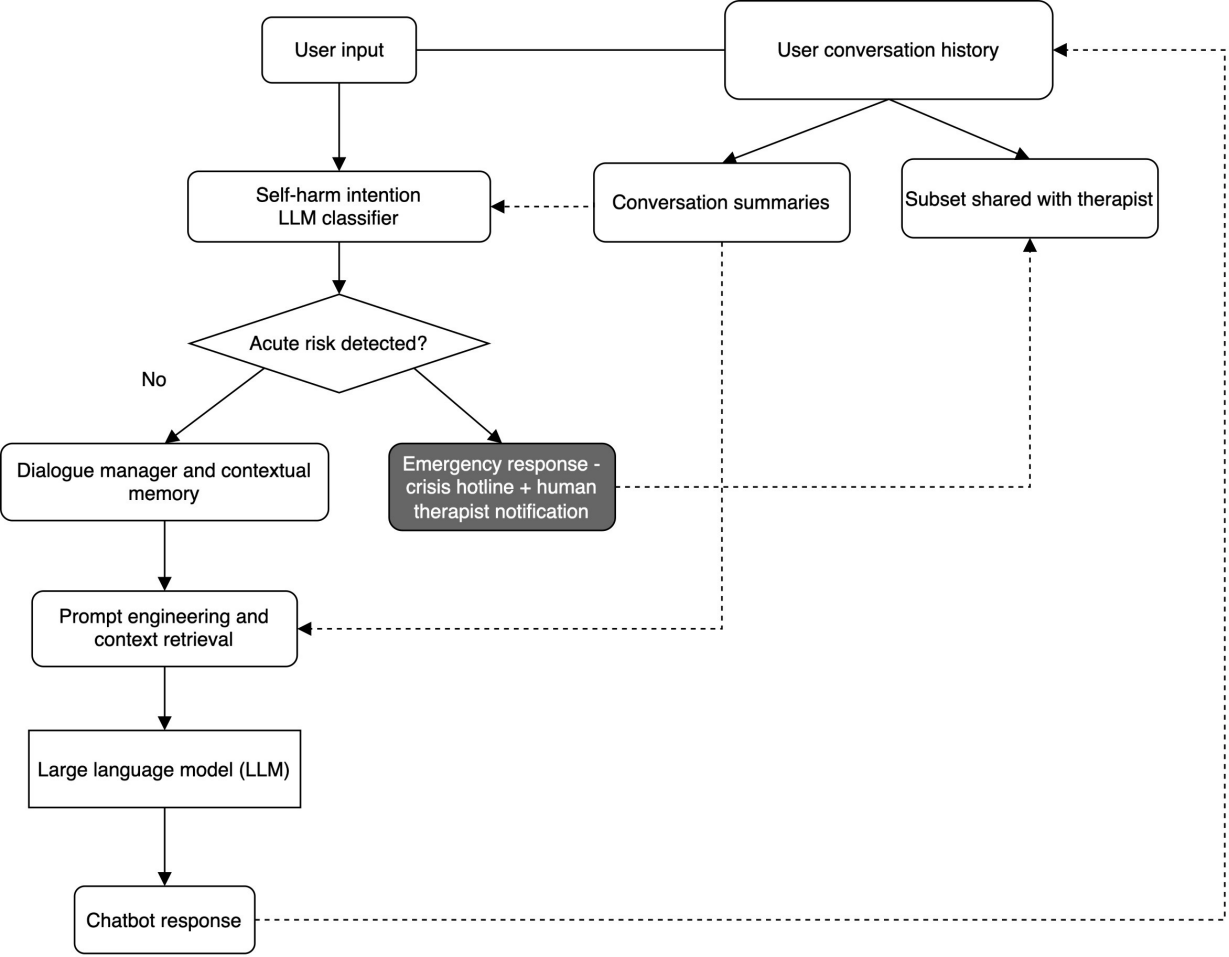
Flow diagram of risk mitigation and escalation protocol.

No serious adverse events occurred in our study, and no participants reported feeling worse due to CBT ChatBot. This mirrors the safety profile reported in similar trials. For example, Heinz et al [50] noted no adverse events in their trial, and users described the Therabot as a positive, supportive influence. Likewise, a recent inpatient pilot [28] observed no incidents when patients used an AI system under supervision, although high-risk individuals (eg, actively psychotic) were excluded. These findings suggest that with proper safeguards, AI chatbots can be introduced into mental health settings without immediate harm. In the current pilot trial, multiple safeguards were implemented to ensure participants’ safety. The CBT ChatBot operated under continuous human oversight, with therapists having the capacity to monitor exchanges and to be alerted if potentially harmful content was generated; however, no such alerts were triggered during the study. We used a clear safety protocol: the AI flagged any mention of self-harm or abuse for immediate human follow-up, sessions included periodic human check-ins, and the chatbot’s suggestions were transparently logged for clinician review (Figure 2). Ethically, users were informed of the chatbot’s limitations—for example, it cannot make diagnoses, it may occasionally produce errors, and it is not a substitute for real-life emergency care. By adhering to emerging best practices (such as the American Psychological Association’s guidelines on AI in therapy and industry standards for AI safety), we can harness the benefits of chatbot assistants while minimizing risk.

Use of the CBT ChatBot was voluntary and explicitly positioned as a supplement to the group sessions rather than a replacement. In addition, all participants were provided with information about external crisis resources beyond the chatbot environment. We suspect that these measures helped prevent misuse and provided users with a sense of security. However, as we scale up, robust ethical guidelines will be essential. Recent research has spotlighted the serious risks of unleashing LLM chatbots as autonomous therapists. Moore et al [52] found that current GPT-based systems (ie, without fine-tuning for psychotherapy purposes) often fail to respond appropriately to high-risk situations. Moreover, Moore and colleagues showed that untrained LLMs used as stand-alone therapists tend to reinforce delusional thinking and user biases rather than challenging them. By being unfailingly agreeable, such AI systems may worsen certain symptoms (eg, feeding into paranoia or grandiosity) and fail to provide the reality testing that is often a crucial therapeutic intervention. Privacy and ethical transparency are also paramount for AI-augmented psychological services. Our participants raised occasional questions about who might see their CBT ChatBot transcripts and whether their data were secure. Similar concerns were also identified in a survey by Alanzi et al [55], in which 67% of patients voiced privacy worries and ethical reservations about AI in therapy. Chatbot providers must ensure compliance with health data regulations and clearly communicate how user conversations are protected. There is also the risk of *overdependence* on AI support. Users may grow to prefer the 24/7 availability and nonjudgmental nature of a chatbot so much that they rely on it instead of seeking professional help when needed. Unregulated use could lead to situations where serious issues (suicidality and trauma) go unaddressed or unrecognized because the person stays within the “closed ecosystem” of a bot that is not equipped to handle a crisis. Considering these issues, *human oversight remains critical*. Heinz et al [50] also emphasized that generative AI systems are not yet suitable to function autonomously in mental health contexts, given the diversity of high-risk scenarios that may arise. Our stance is firmly that the chatbot should complement, not replace, a human therapist.

### Emerging Role of Chatbots and Their Limitations

The present findings add to a growing consensus that generative AI chatbots can serve as a valuable adjunct in mental health care. Rather than viewing these systems as stand-alone therapists, it may be most productive to deploy them as part of a *blended care model*—where the chatbot extends the reach of a human clinician. In this hybrid model, the CBT ChatBot was never intended to serve as a stand-alone therapeutic agent. Instead, its role was strictly supportive, functioning as a digital bridge to ensure that the core UP principles taught by the facilitators were practiced in the participant’s daily environment. In our university context, the CBT ChatBot effectively became a 24/7 “coach” that reinforced the UP techniques students learned during the group counseling sessions. This highlights a key advantage of LLM chatbots: they are instantly accessible and infinitely scalable. A single AI can support hundreds of users simultaneously, even at inconvenient times, providing guidance or a compassionate ear when human providers are unavailable. This could help bridge gaps in care, especially for young adults who often struggle to access traditional therapy due to cost, scheduling, or stigma. Indeed, a recent report noted that affordability-related barriers to health care access are highly prevalent, with individuals experiencing greater psychological distress reporting disproportionately higher rates of such barriers [56]. Chatbots might serve as a lower-barrier first line of support—for example, a student experiencing heightened stress or loneliness could turn to the chatbot for coping strategies and emotional support during nights or weekends, potentially preventing escalation of distress. Additionally, LLM chatbots may function as triage or *stepped-care tools* in public health. They could help monitor symptom changes and alert human clinicians when higher-level intervention is needed. For example, Kuhail et al [20] found that therapists were largely unable to distinguish between transcripts of sessions involving a relational AI chatbot and those involving human therapists and rated the AI-generated interactions as slightly higher in quality, suggesting potential for chatbots to provide preliminary support in contexts of limited mental health resources. Our findings also hint at specific therapeutic roles: the chatbot particularly aided in emotion expression and homework completion—tasks that augment therapy by encouraging daily practice.

In the realm of primary mental health, chatbots such as Wysa and Woebot paved the way with scripted interactions, and now LLM-based systems offer even more dynamic engagement. As LLM technology continues to improve (with better empathy simulation, more accurate knowledge, and multilingual capabilities), we expect its role in mental health support to become more prominent. They might be especially suited for *preventive interventions* and *subclinical populations*—exactly the focus of our study—where the goal is to provide accessible help before problems worsen. By offering a “friendly ear” and evidence-based advice at scale, these chatbots could reduce burdens on counseling centers and reach individuals who otherwise would go without help. Summarizing, the emerging picture is that LLM chatbots are not a panacea or replacement for therapists but they represent a powerful new tool in the continuum of care. Used wisely, they can complement human providers, extending support to times and settings where humans cannot be present.

### Limitations

Despite the promising results, our study—and the current state of AI chatbots—has important limitations. First, as a *pilot trial,* our sample size was small, and participants were not randomly assigned to conditions. This raises the risk of bias: for example, those who chose to use the CBT ChatBot might have been more motivated or tech-comfortable, which could partially explain their improvements. Crucially, since this was a single-arm trial, we cannot separate the therapeutic effects of the human-led group sessions from those of the CBT ChatBot. The observed improvements in generalized anxiety and well-being likely result from a synergistic effect, but they could also be driven primarily by the group therapy component or by nonspecific factors such as participation in a research study. Nonspecific factors such as *expectancy effects* (enthusiasm for a novel technical tool) or the additional attention given to the CBT ChatBot group might have also contributed. The “novelty effect” is a significant concern in digital health, where the initial excitement of interacting with a sophisticated LLM may temporarily boost user motivation and perceived benefit [57]. This enthusiasm often diminishes as the “newness” of the technology fades away, which may explain why some participants eventually found the bot’s responses “generic or repetitive” toward the end of the 4-week period. Furthermore, our sample consisted of university students who may have had high baseline expectations for AI-enhanced support. These expectancy effects (ie, the psychological benefit derived from the belief that a high-tech intervention will be effective) can lead to inflated self-report scores.

Therefore, our outcome findings are preliminary and hypothesis-generating, not confirmatory. We have interpreted the efficacy signals cautiously, using them primarily to estimate effect sizes and refine the intervention for a future definitive trial, rather than to make clinical claims. Another limitation is the *short duration* of the intervention and the lack of follow-up measures. We assessed outcomes immediately after the intervention, but we do not know whether gains were persistent over time. User engagement with digital tools often declines over time; it is therefore plausible that, in the absence of novelty effects and research oversight, students may discontinue using the chatbot after several weeks, which could limit the sustainability of its benefits. Long-term follow-ups (6-12 months) are needed to evaluate whether LLM-based support yields lasting improvements. Our sample was also limited in scope—primarily subclinical young adults at 1 university. This affects *generalizability*. The CBT ChatBot may need further personalization for other populations: for instance, older adults or those with severe mental illness who might have different usability challenges or safety risks. We deliberately focused on a low-risk group; therefore, our positive findings may not generalize to higher-risk settings. Current evidence suggests that human intervention is indispensable for high-risk cases. Additionally, cultural factors could influence chatbot acceptability—our chatbot communicated in Romanian and assumed a relatively informal, friendly tone that suited our sample, but different languages or cultural contexts might require nuanced adjustments (as seen in Kang and Hong’s [51] cross-lingual design addressing Korean cultural needs). Technological limitations of the LLM itself are another concern. While Claude 3.7-Sonnet is currently an advanced LLM, it can still produce errors (“hallucinations”) or overly generic advice despite the layer of CBT consistent system prompts provided in its knowledge database. We encountered a few instances where the CBT ChatBot offered somewhat formulaic responses. Some participants wished the AI could offer more personalized guidance, echoing a common critique that current models, despite being adaptive, may lack the depth of insight of a trained human therapist. These are inherent limits of the technology that could impact efficacy and alliance—for example, if a user senses a response is canned or slightly off target, it could diminish their engagement. In our study, such instances were rare, but as we scale up to more users, ensuring response quality and individualization will be important. Another significant limitation was that we did not formally measure certain relevant outcomes. Notably, the absence of a validated therapeutic alliance measure represents a significant methodological gap, particularly given the central role that alliance, empathy, and rapport play in the theoretical framing of this study. Future research should treat alliance assessment as a primary outcome when evaluating AI-augmented interventions. In particular, including validated alliance measures in future trials would allow for direct comparisons between AI-to-human and human-to-human therapeutic alliance, providing critical insight into the relational dynamics that distinguish AI-augmented from purely human-delivered interventions. Similarly, objective metrics of functional improvement were not included (eg, academic performance and sleep quality). We relied on self-report questionnaires and qualitative feedback. Finally, from an implementation standpoint, there are unresolved questions about *liability and ethics* that lie beyond the scope of our pilot but will need attention. For instance, if a chatbot gives harmful advice or fails to prevent a crisis, who is responsible—the developers, the clinicians deploying it, or the institution? Regulatory frameworks (US Food and Drug Administration, Health Insurance Portability and Accountability Act, etc) for AI in health care are still evolving. Until clear guidelines are in place, institutions may be hesitant to fully integrate such tools. We navigated this by keeping the chatbot in a research setting with clinician oversight, but a real-world rollout would demand rigorous testing, training for providers, and perhaps limiting the chatbot’s autonomy in sensitive situations. In summary, our pilot underscores both the potential and the limitations of LLM-assisted therapy. The approach appears feasible and acceptable, and it shows promise in improving clinical outcomes for the right users. Yet, significant work remains to address the biases, safety concerns, and practical constraints before this can become a standard component of care. We view these limitations not as deterrents but as design challenges for the next phase of research.

### Final Considerations and Future Directions

This study provided an important initial look at how an LLM ChatBot can augment an evidence-based therapy (UP) protocol for young adults. In many ways, the findings offer *proof-of-concept* that such a hybrid intervention is workable: students used the CBT ChatBot, liked it, and benefited from it. These insights now inform the planning of a definitive RCT. A future RCT could be designed to rigorously evaluate the CBT ChatBot’s efficacy and safety on a larger scale, incorporating the lessons learned here. For example, we could aim to recruit a sufficiently powered sample and include appropriate control conditions (eg, UP therapy with vs without the CBT ChatBot) to isolate the chatbot’s added value. We could also incorporate follow-up assessments to see whether improvements are maintained over longer periods. Based on our pilot, the RCT could emphasize *continued exploratory objectives* such as optimizing user engagement strategies and examining potential moderators of outcome (who benefits most from the AI assist?). Our future trials could also incorporate more robust safety monitoring (eg, real-time keyword flags for risk content), a refined consent process addressing privacy concerns, and more specific training for users to maximize effective use of the CBT ChatBot. We could also plan to formally measure mediators such as emotional expression and homework compliance to test the mechanism suggested by Yokotani et al [53] (ie, the chatbot’s encouragement of negative emotion expression leads to symptom relief). The implications of this work extend beyond our campus. If an AI-augmented UP program proves effective, it could be scaled up as a public health intervention for youth mental health. University counseling centers, which frequently operate under considerable resource constraints, might deploy chatbots as a first-line resource for students with mild symptoms or as an after-hours support system. This could free up human counselors to focus on higher-risk cases, thereby optimizing resources. On a broader scale, accessible chatbots might help reduce the incidence of subclinical issues developing into full disorders by providing timely coping tools and psychoeducation. However, we reiterate that any such implementation must proceed carefully, evaluating outcomes in real-world settings and ensuring ethical standards. Our findings contribute to a cautiously optimistic view that LLM chatbots, when thoughtfully integrated, have the potential to “dramatically increase access to support” and serve as a valuable complement to traditional services. As far as we understand these tools now, they will not replace therapists—but they might become trusted assistants, much like a self-help app, only far more interactive and personalized. Ultimately, the goal is to harness technology to reach more people with quality mental health support. The early successes reported (high engagement, symptom reduction, and user satisfaction) are heartening, but they must be balanced against the clear need for continued research and oversight. Our study was an early step, and like other pilots in this nascent field, it had a limited scope. Therefore, we can assert that the integration of an LLM-based chatbot into UP therapy for subclinical students seems feasible, acceptable, and potentially effective in reducing distress. This aligns with other recent pilot and RCT findings that AI chatbots can be efficacious, scalable, and well-liked tools in mental health care. At the same time, significant challenges around safety, ethics, and long-term impact remain. By adhering to high standards (both scientific and ethical) and following a staged research approach (ie, pilot trials, RCTs, and implementation studies), we can systematically address these challenges. If successful, this line of work could contribute to a new paradigm of technology-enhanced psychotherapy—one where human clinicians and AI systems work in tandem to expand the reach and effectiveness of mental health services. We are mindful of the risks but also motivated by the possibility that, with proper care, generative AI could well become a valuable complement to traditional mental health services, helping to fill gaps in care and provide timely help to those in need. This study offers cautious optimism that we are moving in that direction.

## Abbreviations

CBT: cognitive behavioral therapy
LLM: large language model
RCT: randomized controlled trial
UP: Unified Protocol
